# Challenges in Multimorbidity Research: Lessons Learned From the Most Recent Randomized Controlled Trials in Primary Care

**DOI:** 10.3389/fmed.2022.815783

**Published:** 2022-02-24

**Authors:** Martin Fortin, Moira Stewart, José Almirall, Priscilla Beaupré

**Affiliations:** ^1^Family Medicine and Emergency Medicine Department, Université de Sherbrooke, Sherbrooke, QC, Canada; ^2^Centre Intégré Universitaire de Santé et de Service Sociaux du Saguenay Lac St-Jean, Saguenay, QC, Canada; ^3^Department of Family Medicine, Centre for Studies in Family Medicine, Schulich School of Medicine and Dentistry, Western University, London, ON, Canada

**Keywords:** multimorbidity, primary care, randomized controlled trials, interventions, review

## Abstract

Multimorbidity has received much attention and there is a growing number of original studies. However, randomized controlled trials (RCTs) have failed to demonstrate effectiveness of interventions aimed at improving outcomes for patient with multimorbidity in primary care. The purpose of this article is to synthesize and analyze the most recent RCTs to identify the factors that may have contribute to the success or lack of success in order to draw lessons to inform further development in intervention research. A scoping review was conducted to include current up-to-date state-of-the-art studies in primary care published from 2019. Nine articles reporting on six RCTs studies were included in the review. The findings were mixed, with primary outcomes showing no differences between intervention and control groups in four of the six but differences in secondary outcomes in all six. All studies involved family practice patients but interventions took place at different sites, and the time between the beginning of the intervention and the time of evaluation of outcomes varied across studies. Authors reported issues regarding the need for training of care teams, the roles and composition of the teams, the selection of patients and implementation barriers of the complex interventions in trying contexts with not enough time for the changes required. The randomized controlled design may not be the best evaluation design given the complexity of the interventions, and alternative designs should be considered in which qualitative components are included. Further attention to outcome measures and to equity issues is recommended.

## Introduction

Multimorbidity, the presence of two or more long-term conditions, has received much attention among decision makers, researchers and clinicians in the recent years (1). Despite a growing number of original studies, randomized trials have failed to demonstrate effectiveness of interventions aimed at improving outcomes for patient with multimorbidity in primary care (2). Most of them have reported neutral effects or mixed results. These studies however offer a valuable source of information to learn from in order to pave the way for future research in this area in primary care. Which are the best interventions to manage these patients? It is a question to which primary care providers are trying to respond. Research is needed to answer it correctly.

The purpose of this article was to synthesize and analyze the most recent randomized trials of interventions aimed at improving outcomes for patients with multimorbidity to identify the factors that may have contributed to the success or lack of success and to inform further developments in intervention research. Special attention will be given to four elements of the intervention: (1) its description and content; (2) the context in which it was deployed; (3) the evaluation design chosen to test the effectiveness, and (4) the intervention's implementation.

## Methods

In order to synthesize and analyze the publications, a scoping review was conducted following the five stage approach suggested by Arksey and O'Malley's (3). We felt that a scoping review was adequate to address our research question. The central research question of this scoping review was: which factors may have contributed to the success or lack of success in randomized trials of interventions aimed at improving outcomes for patients with multimorbidity?

To identify relevant studies, we used a collection of publications on multimorbidity from the International Research Community on Multimorbidity website (4). The reference list of articles on multimorbidity that we call “Library of publications on multimorbidity” is a document that has carefully and gradually been built for more than 10 years by the Research Community which comprises 71 international researchers as contributors. The articles that have been included in the repository over the years come from different sources that include the databases MEDLINE, SCOPUS, and CINAHL, articles found in the reference lists of published papers, and work communicated by colleagues and other authors. In recent years, it has been regularly updated with searches in MEDLINE three times a year. It was quoted as a source of information in a systematic review (5) and in a large study on multimorbidity (6). It is therefore considered a comprehensive collection of articles on the subject but, as in any collection or review, the absence of some articles is possible. At the time of conducting the search, the collection covered articles published until August 2021. For this review, relevant studies were randomized controlled trials (RCTs) of clinical interventions aimed at improving outcomes for patients with multimorbidity and review articles on the same subject. Review articles were used to identify publications that might have escaped our search of randomized trials.

Our scoping review was not intended to be exhaustive, but we wanted to include enough research papers to answer robustly the research question and to analyze the papers in a way that goes beyond the conclusion of a systematic review by reflecting on the process and mechanisms associated with the effect or absence of effect of the individual trials included. Our intention was more exploratory and explanatory. Ultimately we wanted to generate hypotheses for use in future research.

We included in our review current up-to-date state-of-the-art studies published from 2019. We limited to the last 2 years on the premise that the most recent studies must have already integrated some lessons from the previous ones. For the study selection, two authors (JA and PB) independently assessed the eligibility of publications. In the screening process, the title and the abstract were first reviewed and, if necessary, the complete article. To be included in the process of charting the data, studies had to be conducted in primary care and report at least the following elements: a description of the intervention in context, the design of the evaluation including the choice of outcomes and issues related to implementation.

For charting the data, two authors (JA and PB) conducted separately a comprehensive reading of the articles and extracted the data into a template generated for this purpose following the guide of Arskey and O'Malley (3). The template included the following main items which were described under the item heading: intervention characteristics, context, evaluation design and results, implementation issues and other relevant information. Main items within the template were further subdivided into sub items. Findings from each article were represented twice within the template as both authors conducted their analysis independently. Meetings were held to compare and adjust the data extracted from each article and, after reaching agreement, findings were merged. For collating, summarizing and reporting the results, co-interpretation of different elements of interventions was conducted by all authors. Two senior authors (MF and MS) took the lead in synthesizing and reporting the results.

## Results

**Figure 1:**
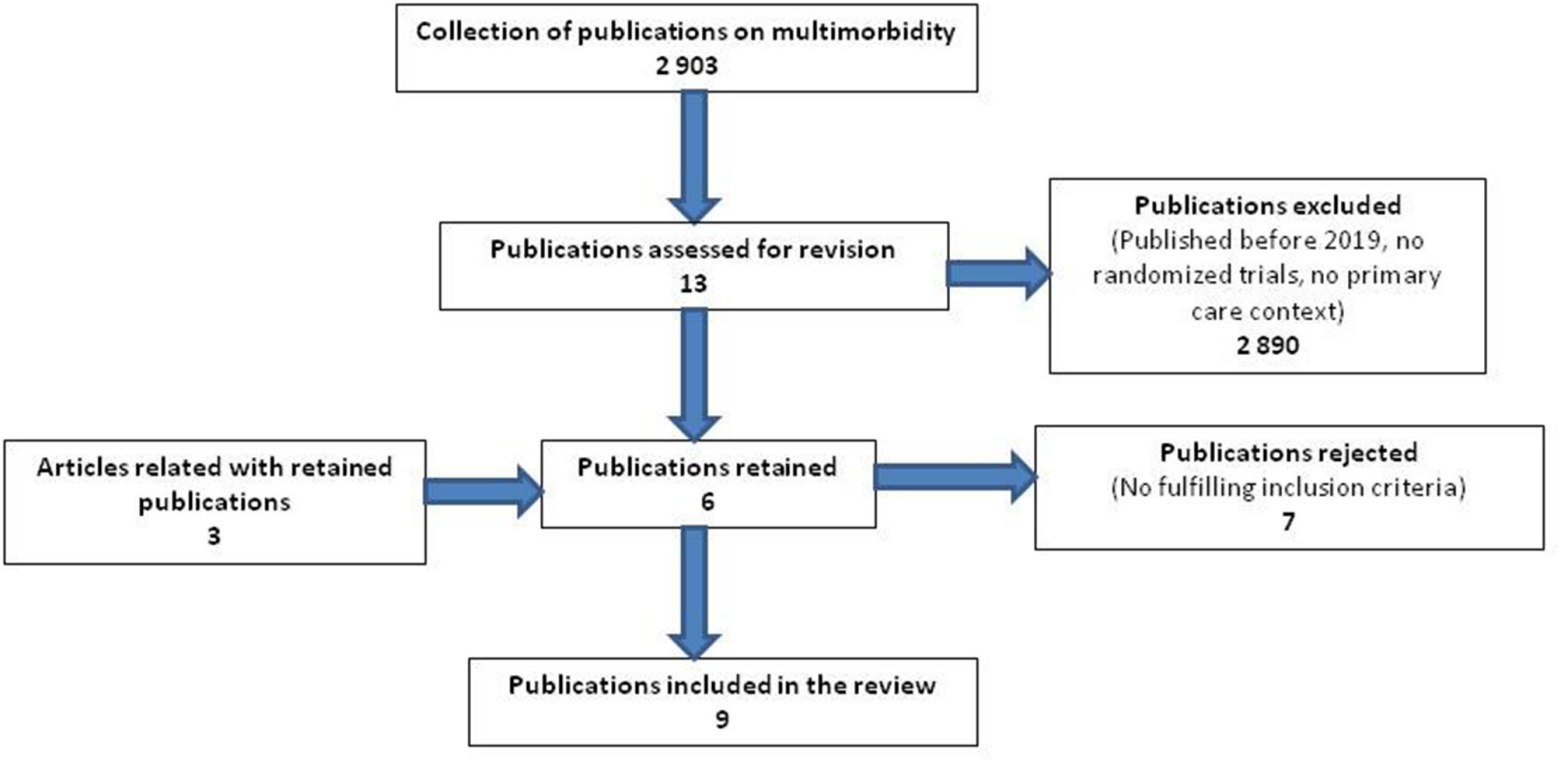
Flow diagram for the selection of publications.

We identified 13 potential articles published since 2019 (Figure 1). Seven articles were rejected for not fulfilling the inclusion criteria. Six articles reporting RCTs studies were included in the review. These six articles were considered the “main” articles of the RCTs studies but, while processing the information from the articles, we learned about another three articles related to these six RCTs, and we included them in the review for a total of nine articles. All reviewed articles are shown in Table 1 grouped by RCT. Summary descriptions of the clinical interventions tested in the included studies are provided in Table 2.

**Table 1:** Articles of RCTs studies of interventions for patients with multimorbidity in primary care settings included in the review.

**References**	**Country**	**Control group**		**Intervention group**		**Methods**		**Results of primary outcomes**
		**Number of patients**	**Mean age, y**	**Number of patients**	**Mean age, y**	**Intervention**	**Primary outcomes^†^**	
**Main article:** Fortin et al. (7)**Related article:**Ngangue et al. (8)	Canada	140	61.1	144	60.8	Pragmatic randomized controlled trial	heiQ SE-CD	Neutral
**Main article:**Stewart et al. (9)**Related article:**Pariser et al. (10)	Canada	77	63.1	86	61.9	Pragmatic randomized controlled trial	heiQ SE-CD	Neutral
**Main article:**Salisbury et al. (6)**Related article:**Salisbury et al. (11)	UK	749	70.7	797	71.0	Pragmatic randomized controlled trial	EQ-5D-5L	Neutral
O'Toole et al. (12)	Ireland	71	65.9	78	65.5	Pragmatic randomized controlled trial	EQ-5D-3L EQ-VAS	Improvement in EQ-VAS in those aged <65 years
Kari et al. (13)	Finland	126	81.4	151	81.0	Randomized controlled trial	SF-36	Neutral
Verdoorn et al. (14)	Netherlands	314	78^*^	315	80^*^	Pragmatic randomized controlled trial	EQ-5D-5L EQ-VAS No. health problems	Improvement in EQ-VAS and No. health problems

* *Median age*.

† *heiQ, Health Education Impact Questionnaire; SE-CD, Self-Efficacy for Managing Chronic Diseases; EQ-5D, EuroQol-5Dimensions; EQ-VAS, EuroQol Visual Analog Scale; SF-36, 36-Item Short-Form Health Survey*.

**Table 2:** Description of included studies.

**References**	**Intervention**	**Change in care delivery**	**Process goal**	**Target patients**	**Setting**	**Theoritical framework**
Fortin et al. (7)	Multifaceted intervention including nurse interview and, consultations with other professionals and individualized care plan.	Professionals were added to existing family medicine teams	To enhance patient self-management	3 or more chronic conditions	7 family medicine groups (FMGs) in Quebec, Canada	Patient Centered Clinical Method (17), Chronic Care Model (16), and Self-management support (18)
Stewart et al. (9)	Multifaceted patient-centered care including a nurse interview at home, a team meeting, a care plan and nurse follow-up	Professionals were added and the team meeting was added	To improve patient engagement in their care and to reduce emergency room visits	3 or more chronic conditions	9 team-based family practices familiar with the intervention in Toronto, Ontario, Canada	Patient Centered Clinical Method (17), Chronic Care Model (16), and Self-management support (18)
Salisbury et al. (6)	Two appointments with a nurse and a named responsible physician, a medication review by a pharmacist, and a collaborative health plan with the patient	Replacing disease-focused reviews of each health condition by a comprehensive 3D multidisciplinary review	To improve continuity, coordination, and efficiency of care	Patients with at least 3 types of chronic conditions	33 practices in England and Scotland	Patient Centered Clinical Method (17), and Chronic Care Model (16)
O'Toole et al. (12)	Professionally-led 6-week group self-management support program	Introducing educational and goal-setting components that included participant interaction and discussion	Self-management support aimed to have a specific focus on function and issues relevant to multimorbidity	2 or more chronic conditions	8 primary care teams in Eastern Ireland	Self-management support (18)
Kari et al. (13)	At-home patient interviews, an interprofessional team meetings (nurse, pharmacist and genral practitionner) to create a care plan	To include in-depth clinical medication and health reviews which are not present within the existing health system	To encourage patient active role in collaborative goal setting and empower them to live well with long-term conditions	Multimorbid patients with 7 or more prescribed medicines	Primary care settings in Tornio, Finland	People Centered Care Model (10), and Chronic Care Model (16)
Verdoorn et al. (14)	Clinical medication reviews (CMRs) with the availability of all clinical data and an extensive patient interview	A CMR review focused on personal goals which is not offered to all patients in usual care	Building on patients' health-related goals and preferences	Community-living multimorbid patients with 7 or more long-term medications	35 community pharmacies in the Netherlands	Patient Centered Approach in Clinical Medication Review (8)

The findings of the six studies were mixed, with primary outcomes showing no differences between intervention and control groups in four of the six. The effects that were found in primary outcomes were: in health related quality of life post intervention (12, 14); and number of health problems (14). Positive impacts of interventions were found in secondary outcomes as follows: in occupational satisfaction and self-reported hospital appointments (12); in mental health outcomes for patients who had depression as well as physical health problems (6); self-reported physical activity (7, 13); healthy eating (7); mental health outcomes in the subgroup with ≥$50K Canadian dollars annual income (with authors highlighting the issue of lack of equity of the intervention) (9); and total number of long-term medications (14).

### Context

All studies involved primary care patients in affiliation with family practices but interventions took place at different sites, which included the family physician's practice (6, 7), face to face or video meetings (9), patients' home (13), and a center where the intervention was delivered (12). They spanned these countries: Canada (7, 8, 10, 15), the United Kingdom (6, 11), Ireland (12), Finland (13), and the Netherlands (14). The wider health policy contexts showed the following features. Some interventions aligned with interests of the Ministries of Health (15); others arose from the interests of local practitioners (13, 14); one had both influences (9). Among the studies, four (6, 7, 9, 12) described in their report a particular context that could have had some influence in the conduct of the intervention. In Quebec, Canada, which has universal health coverage, there was a major reform of healthcare organizations and governance at the time of the intervention, that may have impeded the deployment of the intervention by slowing the decision processes. Several movements of professionals from one site to another and changes of role among managers also challenged and delayed the implementation. In Ontario, Canada, which also has universal health coverage, there was variable access to interprofessional teams to assist with complex health issues; <1-fourth of the population had access to team-based primary care (9, 10), implying a variety of contexts in which the intervention was implemented. However, the policy context in Ontario supported the roll-out of the intervention because of its focus on high users of the health system (the complex, multimorbid patients). In the United Kingdom, which has universal health coverage, the trial was conducted at a time when many practices were under huge strain and struggling to provide essential care, practices were facing other organizational changes, and several of the practices in the trial were facing problems with recruiting physicians (6, 11). Ireland has a mixed public and private primary healthcare system, with one-third of the population entitled to free primary care based on low income; primary care remains underdeveloped and fragmented (12). In summary, the wider practice and health policy context has had positive and detrimental impacts on the research reviewed.

### Evaluation Design

All studies had in common that they were randomized trials. However, the time between the beginning of the intervention and the time of evaluation of outcomes varied across studies. Two studies (7, 9) collected outcome data at baseline and 4 months after the intervention. In one study, outcomes were collected at baseline and 9 and 15 months after recruitment (6). In another, assessments were conducted at baseline, immediately post-intervention at 6 weeks, and after a 6-month follow-up (12). In one of the studies outcome measures were collected at baseline, 3 and 6 months (14). The longest follow-up occurred in a study with assessments at baseline, after 1 and 2-year follow-up (13).

Primary outcomes used in all studies were generic, namely, quality of life (assessed with EuroQol-5 Dimensions; EQ-Visual Analog Scale, EQ-VAS; or the 36-Item Short-Form Health Survey, SF-36) (6, 12–14), health education impact (assessed with the Health Education Impact Questionnaire, heiQ) (7, 9), self-efficacy (assessed with Self-Efficacy for Managing Chronic Diseases, SE-CD) (7, 9), and number of health problems (14). Results in primary outcomes in the studies were neutral, except in one study in which modest improvements were observed in EQ-VAS and the number of health problems (14). Authors suggested that outcome measures aligned neither with goals of the intervention nor patient expectations of the intervention, which authors suggested were mental health, function and feeling validated (9). Studies chose a large number of secondary outcomes which may lead to false positive findings (6, 11).

Some studies used mixed-methods, triangulating a quantitative trial with views of patients, family members and health professionals (6, 7, 9). Two studies also included in their evaluation a qualitative assessment of patients' experience (7, 9).

Selection of patients was an issue in all studies. There were different methods for the selection of patients and for applying inclusion and exclusion criteria before randomization. Four of the studies reported that patients were referred by the family physicians based on their clinical judgement (6, 7, 9, 12). The authors of these studies discussed some limitations associated with this method of selecting patients: (1) as the recruitment was under the control of the primary care providers within the practices, some may have selected patients with lower needs for an intervention on the basis of their motivation leading to baseline scores with little room for improvement (7); (2) only a third of invited patients agreed to participate and this raises the possibility of recruitment bias (6, 11); (3) the sample was unrepresentative of the general population (9). In one study (13), patients were selected at random from a health center database; the response rate was 39%, and this probably led to a selection bias toward older people more willing and able to participate. Another study recognized its failure to include the needy, the avoiders and those with low health literacy (14) implying a lack of equity in the conduct of the research; and (4) the original power calculation was revised downwards because of recruitment difficulties (12).

The comparison group to the intervention was typically usual care. Authors noted that usual care may have been particularly strong, including strategies in the intervention and showing positive effects on outcome (6, 9). Blinding was not possible in these trials with the possibility of professional contamination in small town locations (13) and patient susceptibility to change behavior due to exposure to the consent process (14).

### Implementation of the Intervention

All authors reported some issues with implementation of the interventions. Fortin et al. used formal training at the beginning but found obstacles such as the complexity of the intervention, the health system reorganization in the province at the time of the intervention, the internal organization of the practices, the lack of compatibility of the intervention principles with some family physicians' philosophy and practice (7, 8).

Stewart et al. reported that patients appreciated receiving a summary of the recommendations from the consultation; however, having ≥6 providers in the case conference was linked to negative outcomes (9). Also, having ≥3 h (vs. fewer hours) of nurse follow-up work within 4 months of the case conference was related to statistically significantly less improvement in primary outcomes from baseline to 4-month follow-up (9). These findings imply that the intervention was varied in its implementation and that these variations made a difference to outcomes.

Salisbury et al. described that the staff was familiar with existing disease-specific templates that they had used for many years, and some found it difficult to adapt to the new 3D template; their unfamiliarity with the template required more of their attention and influenced their consultation style in a way which mitigated against the patient-centered approach intended (6). They also mentioned that in some practices, not all general practitioners agreed to take part in the trial, and some participating patients had to consult a different doctor from the one they usually saw, affecting the continuity of care for some patients (6). Although three-quarters of patients received at least one 3D review during the 15-month follow-up period, only about half received the two reviews that were planned (6). Furthermore, an important issue that affected implementation was the wide variation between practices in the roles and competencies of the practice nurses. Some nurses were trained to work only with specific long-term conditions and did not feel confident working with patients with other conditions (6).

Kari et al. discussed that implementation of the people-centered care model into primary care organizations (in order to provide comprehensive, preventive and demand-oriented care for patients), required a shift from providing disease-specific care to people-centered care, which may have been time-consuming (13). Also, probably, it would have been necessary to better identify patients most likely to benefit from this kind of care intervention. They noted that usual care may also have changed during the trial (13).

Verdoorn et al. (14) mentioned the inclusion of training at all stages. They also raised the possibility that patients in the control group could have been prompted to consider obtaining advice about their medication, health problems, or goals by participating in this study (14). In addition, they discussed that when unrealistic or unsolvable goals are proposed by the patient, this may have led to disappointment and a reduction in quality of life. One cannot exclude the possibility that some of the goals may not have been realistic (14). It remained difficult to demonstrate which part of the complex intervention contributed to the observed positive effects (14).

### Synthesis of Results

A synthesis of these six studies revealed four key issues that facilitated implementation or were barriers. First, the importance of training at the beginning of the project was stressed (7) as was the need for ongoing further education (14). Second, the roles and composition of the interdisciplinary care team can be a facilitator to implementation or a barrier. The opportunity for team members to focus on their roles was seen as an asset (7). The wide variation in team members' roles from one practice to another impeded the smooth roll-out of the intervention (6) and a large number of professionals was a detriment to effectiveness (9).

Third, the interventions were complex but personalized which was an asset (14). The personalized aspect aligned with provider values and the coordination in combination with the personalized aspect was appreciated by patients (9). The integration of care models [such as the Chronic Care Model (16) and the Patient-Centered Clinical Method (17)] with specific therapeutic approaches [such as self-management (18) and motivational interviewing (19)] was considered a facilitator of successful implementation (7). However, on the other hand, the new way of practice contrasted with the traditional disease-specific guidelines and that contrast was a potential barrier to speedy change (6).

Finally, several reasons were proposed for inadequate intervention delivery. The context of widespread stress and change in the health care system affected intervention fidelity (6, 7). The short duration of time to mount the intervention and change primary care practice (typically ~4 months) may have been a barrier (6, 7). Health care professionals' values and current practices were an asset if they aligned with the intervention (7, 9) but were not an asset to the intervention roll-out if they conflicted with the intervention, e.g., Salisbury (6) posited that the intervention may have interfered with the previous patient-centered approach of the health professionals and Verdoorn (14) posited that intervention goals may have been considered “risky” and “unrealistic.”

## Discussion

In this review, we sought to identify the factors that may have contributed to the success or lack of success of trials for multimorbidity in primary care in order to draw lessons and to inform further developments in intervention research. The field is indeed heterogeneous, and our intention was humble in writing this paper. Our contribution is to stimulate the conversation and generate new ideas about the research and about the clinical care. The six studies included represent a variety of interventions with enough substance to inspire such conversation.

All multifaceted interdisciplinary interventions involved multiple primary care providers from various disciplines. Evidence supporting such interventions for multimorbidity is scarce, as typically, interventions have shown mixed results (2). But interdisciplinary interventions are in line with the most recent recommendations from NICE (20). However, building such interventions requires close discussion with high level decision makers in order to keep the intervention aligned with policies already in place in order to prepare for scaling-up effective interventions. There are ongoing primary care reforms in many jurisdictions focusing on interdisciplinary work on which it is possible to capitalize and test innovative interventions while embracing the reform.

Implementation and sustainability of intervention deemed to be effective represent a challenge especially when the intervention implies a change in care delivery by a team. We already discussed the importance of aligning the intervention to the policy context to avoid navigating countercurrents. Organizing teamwork in primary care goes beyond having different disciplines working in the same environment. In a previous paper, we suggested an evidence-based framework for effective interventions for multimorbidity in primary care (Patient centered Innovations for Persons with Multimorbidity Framework for effective intervention or PACE in MM Framework) (15). The framework encompasses five components: (1) shared philosophy among the team members; (2) a special attention to the internal relations among the team members including the patient; (3) building on existing external relations within the health care system and the community; (4) professional training of the team members in order to develop integrated care skills; and (5) probably the most important component of enhanced relationship with patients. This framework supplements the classical Chronic Care Model, that was inspirational for most of the studies included in this scoping review, by identifying the processes to create productive interactions between the providers and the patient leading to improve outcomes. It was not clear if all components of this evidence-based framework were enacted in the studies included in this review, but future interventions should consider using this framework in addition to those guiding the specific interventions.

Randomized controlled trials are classically considered the best evaluation design for testing interventions in medical sciences (21). Given the complexity of the interventions within an already complex primary care system, is RCT the best design or is it somewhat limited? Is an RCT appropriate for an intervention that varies from one patient to the other, as opposed to a standard clinical trial looking at a simple intervention for example testing the efficacy of a drug in a specific condition? Most RCT focus on disease-oriented outcomes which is not appropriate in interventions for multimorbidity. In this review, Quality of Life (QofL) was a primary outcome in most studies. This really make sense given the strong association between multimorbidity and low QofL (22). But are the measures of QofL enough sensitive to change to be useful in trial on multimorbidity? This is questionable particularly in trials that are limited in time (6). Authors have suggested a core set of outcomes for interventions in multimorbidity (23). The majority of the studies, included in our review, have used some of these outcomes and reported neutral effects. It is possible that health interventions generate benefits that fall outside the outcomes measured and therefore were not captured in the studies. Qualitative assessment conducted in some of the studies may support this hypothesis (7, 9). New measures are needed to reflect outcomes that are important to patients and sensitive to change to detect benefit from interventions in primary care (6, 7, 24); authors suggest that these include patient function and mental health. Even when the goals of patient are elicited, which is expected for patient-centered interventions, there are no means to ensure that this will be captured in the outcomes given that all patients do not share the same goals. Valid measures of goal attainment to be used on an individual basis are lacking.

A quasi-experimental design with repeated measures where the patients are their own controls may offer an interesting alternative that is more inclusive and a robust enough design that could be included in a systematic review (25). Stepped wedge design could also be considered if the intervention cannot be implemented in all practices at the same time and a control group is not acceptable (25). Researchers have expanded their evaluation designs to include other components in addition to the RCT like qualitative research, process evaluation that could generate explanations and new hypotheses (6, 7, 9).

Selection of patients is of special concern and is prone to bias. Patients who could have most to gain from interventions may be under-represented in participants particularly if the recruitment involves a decision from the primary care provider. Offering patients with high needs to take the risk of not receiving an innovative intervention (thought to be effective) if randomized to the control group, may explain some reluctance from the primary care providers to even offer the intervention. This could explain the low rate of referral or participation in some studies (6, 7, 14). However, this bias also has implications for equity in multimorbidity research.

Conducting a trial in primary care where natural patient-centered interventions are already occurring may reduce the chance of obtaining an effect. It may be that the usual care is already good enough and that enhancing the care may not result in better outcomes. Some of the trials where the baseline evaluation showed already acceptable measures may support this potential explanation (6, 7, 12). None of the studies included in this review specifically included patients with low scores of any outcome measured at baseline.

Implementing changes and conducting pragmatic trial in real world environment may be a bit disruptive for the primary care teams as shown in studies where the fidelity of the intervention was questioned (6, 7). Primary care practices are complex organizations where things could get out of control easily in many circumstances: sick leave of primary care providers, outbreak of infectious diseases, changes in governance, or just the chaos of normal days working with sick peoples with high needs. Studies in which the practices which had contributed to the intervention seemed to have fewer implementation issues than when the intervention was suggested by researchers or others outside the practices.

The question of equity is of special concern. Two indications of lack of equity deserve attention: one in the effect of the intervention only for higher income groups of patients (9); and a second in the selection of patients by inadvertently failing to include the needy, the avoiders and those with low health literacy (14). Our interventions and our research must thoughtfully address equity issues raised here.

This scoping review has limitations. The search was limited to the most recent years which, while a limitation, could also be considered an asset as these recent studies were seen to have learned from the previous ones and represent the most current state-of-the-art studies. The goal of this scoping review was never to be exhaustive in the identification of studies but to include enough papers to be able to make suggestions for the future of interventional research on multimorbidity in primary care. There were enough commonalities among the studies to support this idea.

## Conclusion

This scoping review identified several lessons on planning for future intervention studies on multimorbidity in primary care. Interdisciplinary teams as the basis for most interventions, while recommended, may need more support by policy and practice leadership to be successfully deployed and evaluated. The randomized controlled design may not be the best evaluation design given the complexity of the interventions; alternative designs should be considered in which qualitative components are included. Special attention should be given to outcome measures ensuring that they are better aligned to patient goals. Selection of patients prone to bias toward the less needy, hampers the ability to document effectiveness and raises question about equity in research. Implementation of the interventions needs special attention and enough time to gel.

## Author Contributions

MF and MS contributed to conception, design of the study, and took the lead in synthesizing and reporting the results. JA and PB conducted the study selection and extracted the data. JA wrote the first draft of the manuscript and co-interpretation of different elements of studies was conducted by all authors. All authors contributed to the article and approved the submitted version.

## Funding

Funding for this review was from the Patient-Centered Innovations for Persons with Multimorbidity in Primary Care, Canadian Institute of Health Research Community-based Signature Initiative. MF was funded by the Research Chair on Chronic Diseases in Primary Care, Université de Sherbrooke, (2007–2020). MS was funded by the Dr. Brian W. Gilbert Canada Research Chair (Tier 1) in Primary Health Care Research (2003–2017).

## Conflict of Interest

The authors declare that the research was conducted in the absence of any commercial or financial relationships that could be construed as a potential conflict of interest.

## Publisher's Note

All claims expressed in this article are solely those of the authors and do not necessarily represent those of their affiliated organizations, or those of the publisher, the editors and the reviewers. Any product that may be evaluated in this article, or claim that may be made by its manufacturer, is not guaranteed or endorsed by the publisher.
